# Supplementary material for: Investigating How Genomic Contexts Impact IS5 Transposition Within the Escherichia coli Genome
Source: Microorganisms. 2024 Dec 16;12(12):2600. doi: 10.3390/microorganisms12122600 (PMC11677980; doi:10.3390/microorganisms12122600)
Supplement: Supplementary file 1 [file microorganisms-12-02600-s001.zip › Figure S1.pptx]

## Slide 1
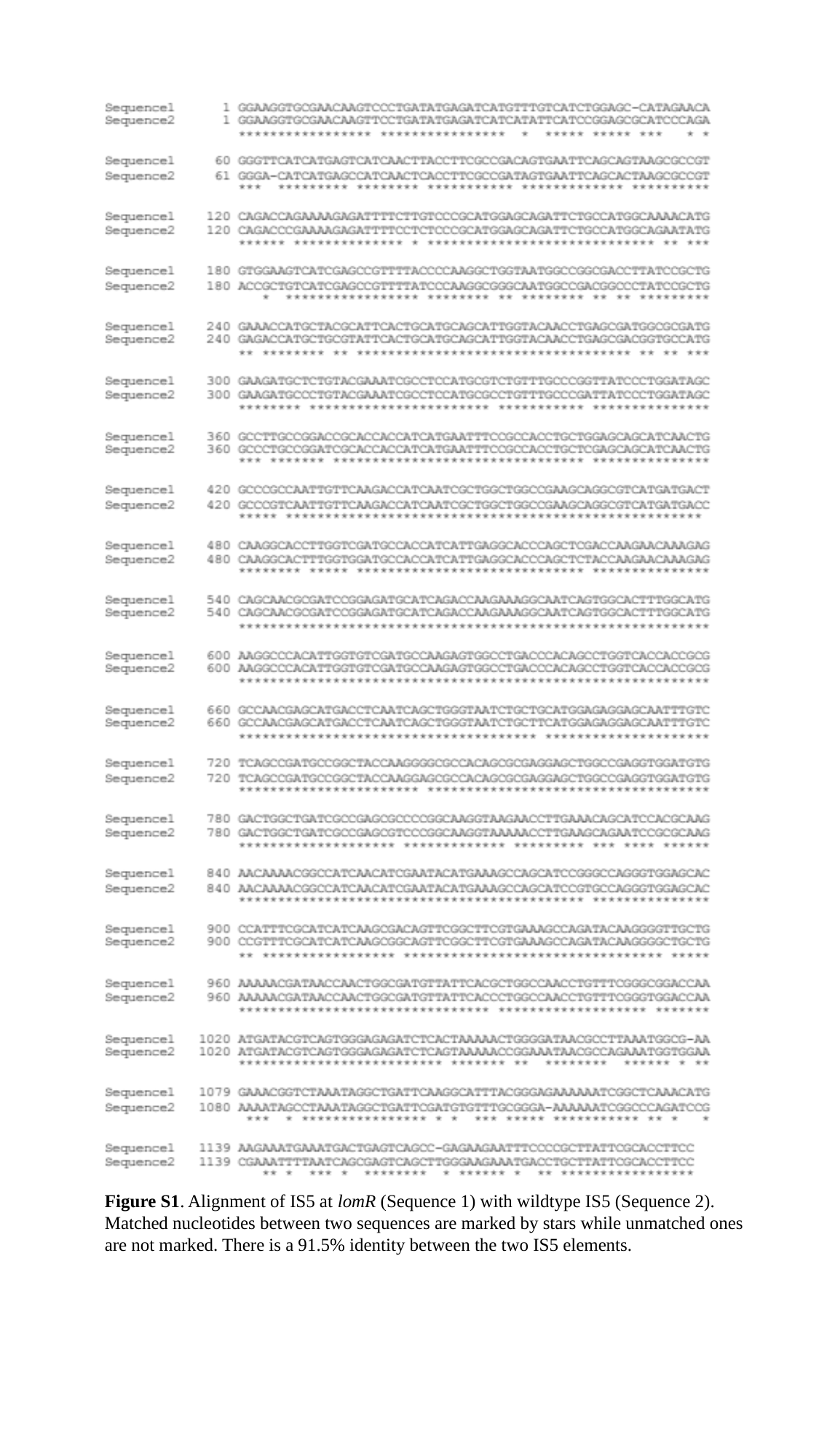

Figure S1. Alignment of IS5 at lomR (Sequence 1) with wildtype IS5 (Sequence 2).
Matched nucleotides between two sequences are marked by stars while unmatched ones are not marked. There is a 91.5% identity between the two IS5 elements.
