# Supplementary material for: Investigating How Genomic Contexts Impact IS5 Transposition Within the Escherichia coli Genome
Source: Microorganisms. 2024 Dec 16;12(12):2600. doi: 10.3390/microorganisms12122600 (PMC11677980; doi:10.3390/microorganisms12122600)
Supplement: Supplementary file 1 [file microorganisms-12-02600-s001.zip › Figure S2.pptx]

## Slide 1
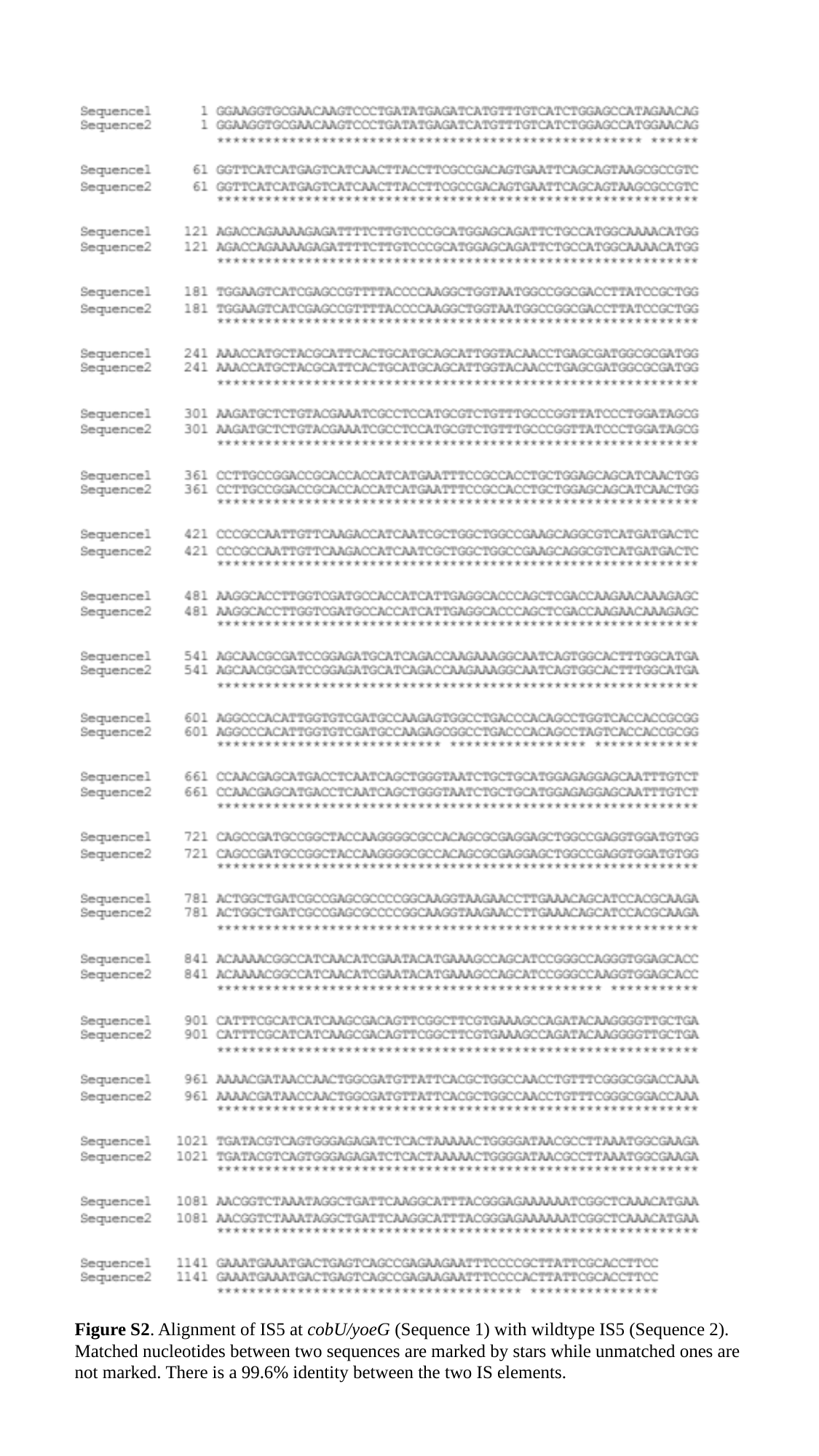

Figure S2. Alignment of IS5 at cobU/yoeG (Sequence 1) with wildtype IS5 (Sequence 2).
Matched nucleotides between two sequences are marked by stars while unmatched ones are not marked. There is a 99.6% identity between the two IS elements.
