# Supplementary material for: Investigating How Genomic Contexts Impact IS5 Transposition Within the Escherichia coli Genome
Source: Microorganisms. 2024 Dec 16;12(12):2600. doi: 10.3390/microorganisms12122600 (PMC11677980; doi:10.3390/microorganisms12122600)
Supplement: Supplementary file 1 [file microorganisms-12-02600-s001.zip › Table S1_strains.pdf]

Supplementary Table 1. Strains used in this study

Strains	Genotype or description	Reference
BW25113	Wild type, *lacI*^{q} *rrnB*$_{T14}$ Δ*lacZ*$_{WJ}$ Δ*hsdR*514 Δ*araBAD*$_{AH33}$ Δ*rhaBAD*$_{LD78}$	[46]
BW-RI	Constitutive expression of *tetR* and *lacI* in BW25113	[48]
ZZ245	ΔIS5 within *ykfC* in BW25113	This study
ZZ246	ΔIS5 within *nmpC* in BW25113	This study
ZZ247	Δ*nmpC* in BW25113	This study
ZZ248	ΔIS5 in the *gltI*/*lnt* intergenic region in BW25113	This study
ZZ249	ΔIS5 in the *ynaI*/*ynaJ* intergenic region in BW25113	This study
ZZ250	ΔIS5 within *wbbL* in BW25113	This study
ZZ251	ΔIS5 within *yejO* in BW25113	This study
ZZ252	ΔIS5 within *yghO* in BW25113	This study
ZZ253	ΔIS5 within *yhcE* in BW25113	This study
ZZ254	ΔIS5 within *yhiS* in BW25113	This study
ZZ255	ΔIS5 at *nmpC*, *wbbL*, *yejO* and *gltI*/*lnt* loci in BW25113	This study
ZZ256	Deleting the *nmpC* promoter (P_{nmpC}) in BW25113	This study
ZZ257	Adding a *rrnB* terminator downstream of P_{nmpC} in BW25113	This study
ZZ258	Substituting P_{tet} for P_{nmpC} in BW25113	This study
ZZ259	P_{tet} driving *nmpC* in BW-RI that constitutively produces TetR	This study
ZZ260	Adding a *rrnB* terminator downstream of *nmpC* (between *nmpC* and *quuD*) in BW25113	This study
ZZ261	Inserting P_{tet} between *nmpC* and *quuD*, driving *ins5CB*	This study
ZZ263	Deleting all active IS5 copy save for one at *nmpC* in BW25113	This study
ZZ264	P_{tet} driving *ins5A* at the *intS* locus in BW25113	This study
