# Supplementary material for: Investigating How Genomic Contexts Impact IS5 Transposition Within the Escherichia coli Genome
Source: Microorganisms. 2024 Dec 16;12(12):2600. doi: 10.3390/microorganisms12122600 (PMC11677980; doi:10.3390/microorganisms12122600)
Supplement: Supplementary file 1 [file microorganisms-12-02600-s001.zip › Table S2_Primers.pdf]

Supplementary Table 2. Oligonucleotides used in this study

Name	Sequence	Use
IS5-ykfC-P1	ccgcaggagaaagccagaaacttcgccgcatcgctgacagcactgttattgtgtag gctggagctgcttc	Replacing IS5 at *ykfC* with *km*^{r} to make ZZ245
IS5-ykfC-P2	tggggattaataatcagatacaaaaagggccgccagctcataacgcctccattccg gggatccgtcgacctg	Replacing IS5 at *ykfC* with *km*^{r} to make ZZ245
ykfC-ver-R	ttgaagaataatcctgtgccaatcac	Verification of *km*^{r} substitution for IS5 at *ykfC*
IS5-nmpC-P1	ggtaaaccagacctacagcaacgatgtcatcagtgcttacaccgagtgcttgtgtag gctggagctgcttc	Replacing IS5 at *nmpC* with *km*^{r} to make ZZ246
IS5- nmpC -P2	actttcgttgattacaaaatcaacctgcttgacaaaaatgacttcactaaattccgggg atccgtcgacctg	Replacing IS5 at *nmpC* with *km*^{r} to make ZZ246
nmpC-P1	tcagattagaactggtaaaccagacctacagcaacgatgtcatcagtgcttgtgtag gctggagctgcttc	Replacing IS5 at *nmpC* with *km*^{r} to make ZZ247
nmpC -P2	aatgaaaaaattaacagtggcaatttctgctgtagctgcatcagtactaatgattccgg ggatccgtcgacctg	Replacing IS5 at *nmpC* with *km*^{r} to make ZZ247
nmpC-ver-R	atgtaggtgcaacctattacttcaac	Verification of *km*^{r} substitution for *nmpC* or IS5 at *nmpC*
IS5-lnt-P1	agtgtttgtgttgttatcgtctgcaactttattgtgcagtgttgtgcctgtgtgtaggctgg agctgcttc	Replacing IS5 at *gltI/lnt* with *km*^{r} to make ZZ248
IS5-lnt-P2	aacaataaaacagcaatatctttacatttagccaaactaaatgttacctaaattccggg gatccgtcgacctg	Replacing IS5 at *gltI/lnt* with *km*^{r} to make ZZ248
lnt-ver-R	tcagtacaacctagttgcaccacag	Verification of *km*^{r} substitution for IS5 in the *gltI/lnt* intergenic region
IS5-ynaJ-P1	cgacaattcattcagatcatcaatagtcagggaaggaagtagcaacattagtgtgta ggctggagctgcttc	Replacing IS5 at *ynaI*/*ynaJ* with *km*^{r} to make ZZ249
IS5-ynaJ-P2	tgaaaacctcacctcttagctggatttacaatcaacgcattgattagttagattccggg gatccgtcgacctg	Replacing IS5 at *ynaI*/*ynaJ* with *km*^{r} to make ZZ249
ynaJ-ver-R	aatcatgatgtaatccttgtgcagac	Verification of *km*^{r} substitution for IS5 in the *ynaI*/*ynaJ* intergenic region
IS5-wbbL-P1	tcctgcgcaccaatcaacaaccgtatcagaatagatactttctttaggaatgtgtaggc tggagctgcttc	Replacing IS5 at *wbbL* with *km*^{r} to make ZZ250
IS5-wbbL-P2	aaatttcctgtgctttctgattttattgtgtcatttatgttagggattaaattccggggatcc gtcgacctg	Replacing IS5 at *wbbL* with *km*^{r} to make ZZ250
wbbL-ver-R	atcatgaagcatgatgatttgctgac	Verification of *km*^{r} substitution for IS5 at *wbbL*
IS5-yejO-P1	actgtctcaccatactcaacagttgatgccaatgctatgggtgaatataatgtgtaggc tggagctgcttc	Replacing IS5 at *yejO* with *km*^{r} to make ZZ251
IS5-yejO-P2	ctgtttctctttgtcgttccgcaatatctgttctggtggctacagcgttatattccgggga tccgtcgacctg	Replacing IS5 at *yejO* with *km*^{r} to make ZZ251

yejO-ver-R	ttcataggatgaaagctcaatgcatc	Verification of *km*^{r} substitution for IS5 at *yejO*
IS5-yghO-P1	gataaagtgatgacggaaaagcgataaatgccttaaggtcatttttattaatgtgtagg ctggagctgcttc	Replacing IS5 at *yghO* with *km*^{r} to make ZZ252
IS5-yghO-P2	tttcgctggtgtttggcgttaagtatctcgaagcgtatgacctgatttagattccgggga tccgtcgacctg	Replacing IS5 at *yghO* with *km*^{r} to make ZZ252
yghO-ver-R	agaccgtggttgttaggtgtgaagtc	Verification of *km*^{r} substitution for IS5 at *yghO*
IS5-yhcE-P1	gccagtggcaaaaatgacaatccgggcagcacaggaacagcatcaattagtgtgt aggctggagctgcttc	Replacing IS5 at *yhcE* with *km*^{r} to make ZZ253
IS5-yhcE-P2	ctttatataatttggggtagggtgttctttatgtaaaaaaatagtgtcacattccgggg atccgtcgacctg	Replacing IS5 at *yhcE* with *km*^{r} to make ZZ253
yhcE-ver-R	ttcgaattgatattcagacatttctg	Verification of *km*^{r} substitution for IS5 at *yhcE*
IS5-yhiS-P1	ggttttatttcgaagatctacttaatataaatttcactgacgcaaatttagtgtgtaggct ggagctgcttc	Replacing IS5 at *yhiS* with *km*^{r} to make ZZ254
IS5-yhiS-P2	ccacttcttgaccattttgcataaacaccagccaattattgggggaagtaattccggg gatccgtcgacctg	Replacing IS5 at *yhiS* with *km*^{r} to make ZZ254
yhiS-ver-R	tccaccaggaccgtcagattgcctg	Verification of *km*^{r} substitution for IS5 at *yhiS*
PnmpC-km-P1	tgaaccttcaaattatagagcacttataaataacagccgttaatataaattgtgtaggct ggagctgcttc	Replacing *nmpC* promoter with *km*^{r} to make ZZ256
PnmpC-km-P2	cattagtactgatgcagctacagcagaaattgccactgttaatttttcatattccgggg atccgtcgacctg	Replacing *nmpC* promoter with *km*^{r} to make ZZ256
nmpC-ver-R2	tatctttggaggaaccttgagattcag	Verification of *km*^{r} substitution for *nmpC* promoter
IS5-GST-R	tcaggttgtaccaatgctgcatgcag	Amplifying and sequencing cDNA for 5’ RACE
nmpC-GST-R	taagtagtatcaccatcatctgcatc	Sequencing IS5/*nmpC* fusion cDNA for 5’ RACE
PnmpC-T-P1	acttacatcttgaaataatcacattgattagatgaatatttatcgcgcagtgtgtaggct ggagctgcttc	Adding a *rrnB* terminator downstream of P_{nmpC} to make ZZ257
PnmpC-T-P2	gtactgatgcagctacagcagaaattgccactgttaatttttcatcgtgaaaggttcat cgcgctcgagacgca	Adding a *rrnB* terminator downstream of P_{nmpC} to make ZZ257
Ptet-nmp-P1	gccggtttaaattgaaccttcaaattatagagcacttataaataacagccgtgtgtagg ctggagctgcttc	Replacing P_{nmpC} with P_{tet} to make ZZ258
Ptet-nmp-P2	ccattagtactgatgcagctacagcagaaattgccactgttaatttttcatggtaccttt ctcctctttaatgaattc	Replacing P_{nmpC} with P_{tet} to make ZZ258
euuD-T-P1	agagatggatatcgttgttaataactctaattaatatgccaattgtttactgtgtaggctg gagctgcttc	Adding a *rrnB* terminator downstream of *nmpC* to make ZZ259
euuD-T-P2	actatataaaccttctgttatatattaccctttatttttgggggcgtctcaaaggttcatcg cgctcgagacgca	Adding a *rrnB* terminator downstream of *nmpC* to make ZZ259
nmpC-ver-F2	tgctgtaggtctggtttaccagttc	Verification of *rrnB* terminator insertion downstream *nmpC*

Ptet-quuD-P1	agggtaatatataacagaaggtttatatagttagaagcaaggttgtgcttctgtgtagg ctggagctgcttc	Insertion of P_{tet} downstream of *nmpC*, driving *insCB*
Ptet-quuD-P2	ctgatgacatcgttgctgtaggtctggtttaccagttctaatctgattacggtacctttct cctctttaatgaattc	Insertion of P_{tet} downstream of *nmpC*, driving *insCB*
intS-km-P1	agatttacagttcgtcatggttcgcttcagatcgttgacagccgcactccatgtgtagg ctggagctgcttc	Amplification of the "km^r:*rrnB*T:P_{tet}" cassette
Ins5A-Ptet-R	gtcggcgaaggtaagttgatgactcatggtacctttctcctctttaatgaattc	Amplification of the "km^r:*rrnB*T:P_{tet}" cassette
Ptet-ins5A-F	gaattcattaaagaggagaaaggtaccatgagtcatcaacttaccttcgccgacag	Amplification of *ins5A*
ins5A-int-P2	aggtcgctcactccaccttctcatcaagccagtccgcccaccattgcatcatttagtg agatctctcccactgacgtatc	Amplification of *ins5A*
